# Destroying Nerves

**Published:** 1872-04

**Authors:** W. H. Shadoan


### SELECTED ARTICLES.

---

### ARTICLE VI.

### *Destroying Nerves.*

BY W. H. SHADOAN, D. D. S.

This operation is condemned by a few operators, as wholly unnecessary. We will agree that many nerves are destroyed that should have been preserved, but there are cases in which I do not attempt to save pulps.

The question under consideration is not when a pulp should or should not be destroyed, but the easiest wav of destroying it and causing the least pain to the patient.

*Preparation of Case.*—The nerve should in all cases be laid bare before the application is made, otherwise there is a liability of great pain for a time at least. It is not always that the patient can bear the pain of the operation; when this is so, the application of chloroform for a short time, or creasote and tincture of aconite will obtund the sensibility until the excavation can be completed.

If the tooth to be operated on has but a single root, the point of exposure need not be so large—half a line in diameter will do; but if it is a molar, then always make the orifice of exposure as large as possible, a full line in diameter, if circumstances will permit. Wound the pulp, if possible, before its excavation. A drop of blood from the nerve of a tooth depletes it sufficiently to allow of its destruction with rarely ever any pain, and after all, that is just what is desirable.

*Material Used.*—I invariably use *arsenious acid* and *carbolic acid* without sulphate or the acetate of morphia. I once used a paste of arsenous acid and sulph morphia, and in nine cases out of ten there was great pain for a considerable time, hence its total abandonment. Now I use only arsenic and carbolic acid.

*Method of Application.*—It matters very little where the application is to be made; whether in an incisor tooth or a third molar, the tooth should be invested with a napkin; dry out the cavity thoroughly with punk, or softly rolled bibulous paper, and with carbolic acid wet the pulp and surrounding portion of the dentine; then the arsenic is applied on the point of a small bit of cotton rolled between the fingers until it is quite firm about a line in diameter, and as long as necessary. (For a front tooth a quarter of an inch, and for a back tooth a half of an inch long, will be required.) This is taken up with the plugging pliers, and dipped into the solution of acid, and the point touched to the dry pulverized arsenic, when a sufficient amount will adhere to the cotton, which is carried directly to the point intended. In this way the application may be made directly on the point of exposure, and never allowed to come in contact with the gum or lip of the patient. A piece of cotton laid on the arsenic loosely is all that is needed to complete the operation. I never leave the cotton used to carry the arsenic to the nerve in the tooth, unless it be a molar, and seldom then. The arsenic should be removed within twenty-four hours, when generally the nerve is sufficiently obtunded to be removed, though frequently the pulp may remain for a few days without molestation, when it can more easily be removed.—*Dental Register.*

---

